# Supplementary material for: In Silico Analysis of a GH3 β-Glucosidase from Microcystis aeruginosa CACIAM 03
Source: Microorganisms. 2023 Apr 11;11(4):998. doi: 10.3390/microorganisms11040998 (PMC10146135; doi:10.3390/microorganisms11040998)
Supplement: Supplementary file 1 [file microorganisms-11-00998-s001.zip › microorganisms-2126059-supplementary.pdf]

In Silico Analysis of a GH3 β-glucosidase from *Microcystis aeruginosa* CACIAM 03

Gustavo Marques Serra [1,*], Andrei Santos Siqueira [2], Fábio Alberto de Molfetta [3], Agenor Valadares Santos [1] and Luciana Pereira Xavier [1,*]

[1] Laboratório de Biotecnologia de Enzimas e Biotransformações, Instituto de Ciências Biológicas, Universidade Federal do Pará-UFPA, Belém 66075-110, Brazil; avsantos@ufpa.br
[2] Laboratório de Tecnologia Biomolecular, Instituto de Ciências Biológicas, Universidade Federal do
Pará-UFPA, Belém 66075-110, Brazil; andrei.siqueira@icb.ufpa.br
[3] Laboratório de Modelagem Molecular, Instituto de Ciências Exatas e Naturais, Universidade Federal do Pará-UFPA, Belém 66075-10, Brazil; fabioam@ufpa.br
* Correspondence: gustavo.serra@icb.ufpa.br (G.M.S.); lpxavier@ufpa.br (L.P.X.); Tel.: +55-91-98182-3213 (G.M.S.); +55-91-99294-4213 (L.P.X.)

Supplementary Material

Table S1. Validation of three-dimensional structure of MaBgl3 using MolProbity, Verify3D and ERRAT.

Ramachandran plot (%)					
Favorable Regions	Allowed Regions	Disallowed Regions	Verify 3D score (%)	ERRAT score (%)	RMSD (Å)
95.19	4.81	0.0	97.32	89.08	0.087

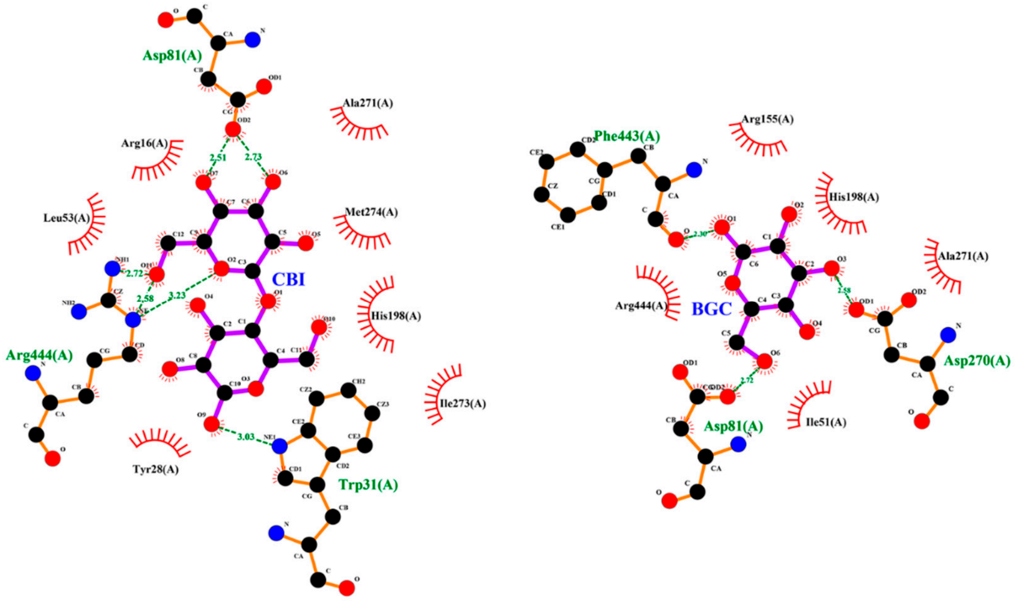

(**a**) (**b**)

Figure S1. Hydrogen interactions were obtained with PoseView server. (**a**) CBI and (**b**) BGC.

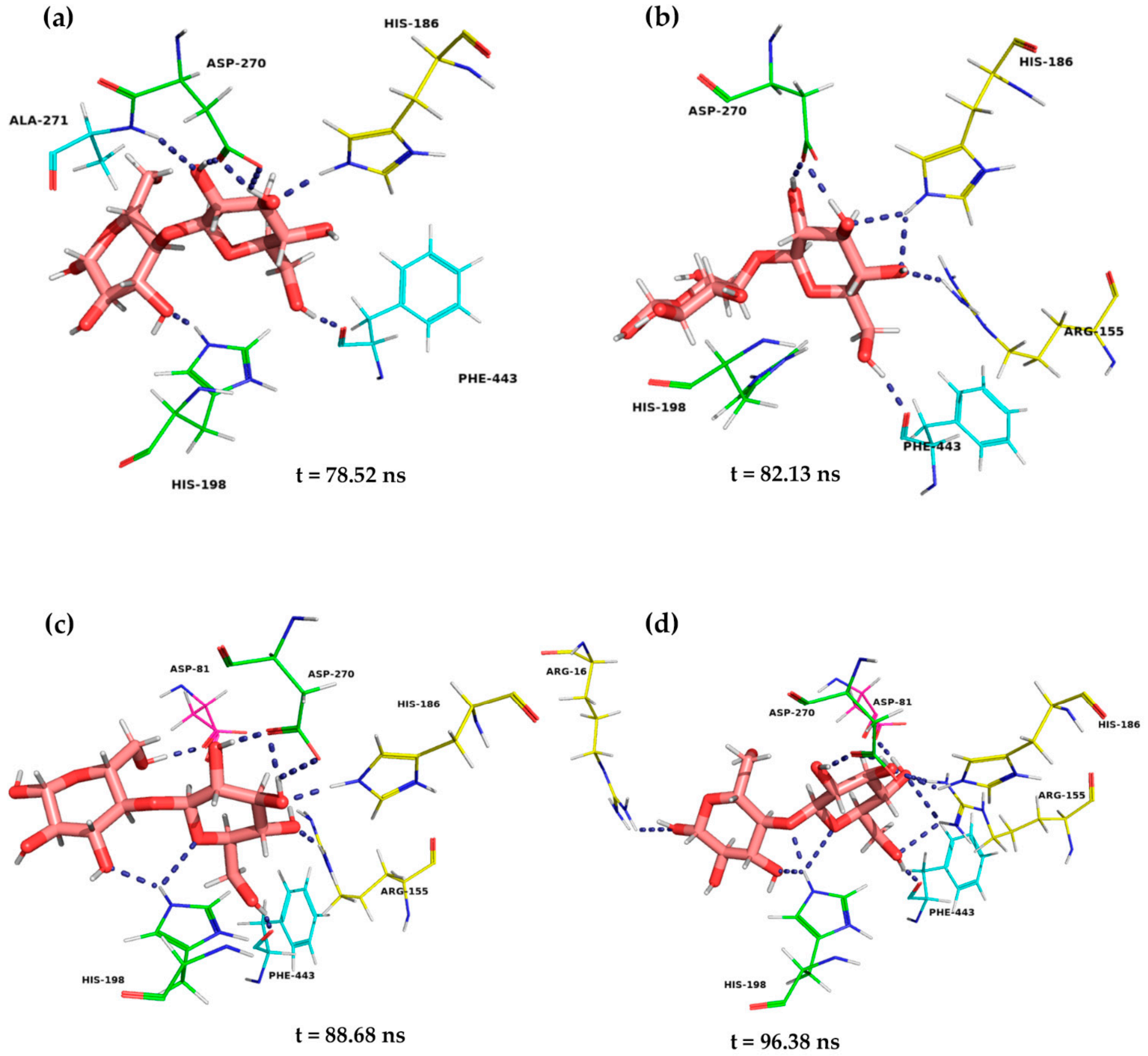

Figure S2. Stick representation of CBI at time (**a**) 78.52 ns, (**b**) 82.13 ns, (**c**) 88.68 ns and (**d**) 96.38 ns. Hydrogen bonds interactions are in dashed blue lines.

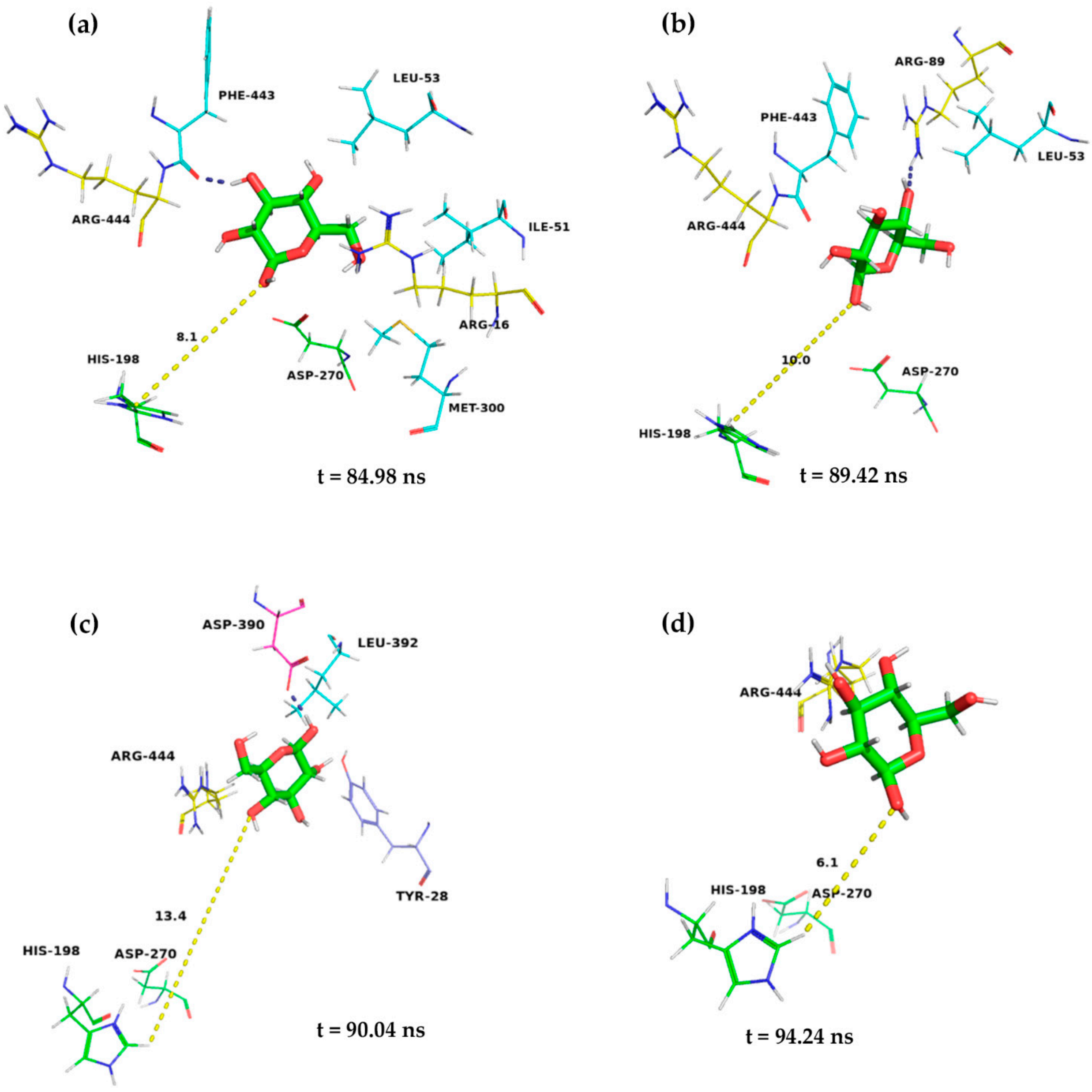

Figure S3. Stick representation of BGC at time (**a**) 84.98 ns, (**b**) 89.42 ns, (**c**) 90.04 ns and (**d**) 94.24 ns. Hydrogen bonds interactions are in dashed blue lines and distance measures are in yellow dashed lines.

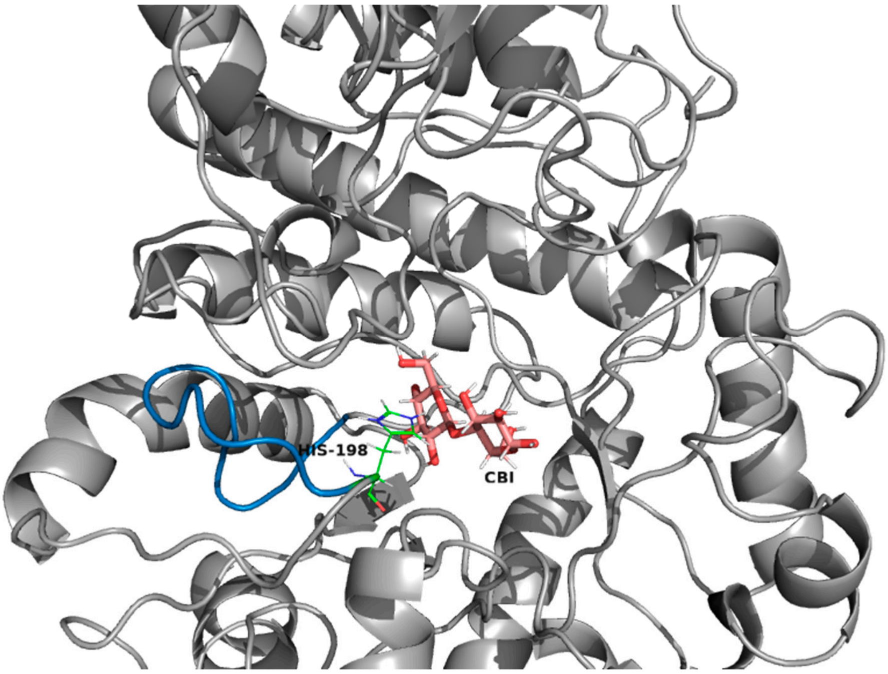

Figure S4. MaBgl3 final pose of trajectory. General acid/base catalyst His198 is present in the loop of the enzyme active site and the substrate CBI.
